# Editorial: Leveraging genomics, phenomics, and plant biotechnology approaches for improving abiotic and biotic stress tolerance in cereals and legumes

**DOI:** 10.3389/fpls.2023.1307390

**Published:** 2023-11-07

**Authors:** Paola Leonetti, Moemen S. Hanafy, Rupesh Tayade, Muthusamy Ramakrishnan, Humira Sonah, Hans- Joerg Jacobsen

**Affiliations:** ^1^ Institute of Sustainable Plant Protection (IPSP), National Research Council of Italy (CNR), Bari, Italy; ^2^ Plant Biotechnology Department, Biotechnology Research Institute, National Research Centre (NRC), Giza, Egypt; ^3^ Department of Applied Biosciences, Kyungpook National University, Daegu, Republic of Korea; ^4^ Bamboo Research Institute, School of Life Sciences, Nanjing Forestry University, Nanjing, Jiangsu, China; ^5^ Department of Biotechnology, Central University of Haryana, Mahendragarh, Haryana, India; ^6^ Institute of Plant Genetics, Section of Plant Biotechnology, Leibniz Universität Hannover, Hannover, Germany

**Keywords:** cereal, legume, genomics, crops, abiotic, biotic, stress tolerance

Climate change poses new threats to plant production as environmental stresses increase significantly. Biotic and abiotic stress that have adverse effects on plant growth and crop productivity and can be major constraints on yield. This will particularly affect major food crops, including legumes and cereals. Biotic and abiotic stresses, such as salinity, drought, chemical toxicity, extreme temperatures and oxidative stress are often interrelated and become serious threats to agriculture. These extreme conditions induce cellular damage and disruption of plant growth and development by a series of morphological, physiological, biochemical and molecular changes. For example, these extreme stresses are often leading to oxidative damage and excessive creation of reactive oxygen species (ROS) in plant cells. Usually, plants interact with these extreme conditions by their innate antioxidative defense system that including enzyme and non-enzyme elements (Passardi et al., 2007). However, in most cases the plants’ innate antioxidant systems is insufficient to detoxify it and protect themselves from the negatives of biotic and abiotic stresses. The most obvious stress symptoms are the losing of osmotic responsiveness and wilting.

Therefore, enhancing the productivity of these important crops is important for the food security of growing populations worldwide. High heterozygosity imposes severe limitations on breeding new varieties of major food crops with desired traits. Conventional plant breeding and genetic research have significantly evolved in recent decades. However, to meet the growing human population’s food demand, especially for major staple cereal and legume crops, genetic gain must be enhanced, making it necessary to exploit genetic diversity within cultivated crops and wild relatives. With modern technologies, plant breeders can use these additional resources of diversity to address problems like biotic and abiotic stresses and yield by combining methods from genomics, and advanced analytics in cereal and legume breeding programs on a massive scale. This topic explores how the recent advances in genomics and plant biotechnology approaches are helping researchers to answer questions related to crop improvement. Therefore, we encouraged contributions to this Research Topic to advance and disseminate knowledge across various facets of this critical field. Our goal is to illuminate the molecular mechanisms that underlie biotic and abiotic stress tolerance, enhance comprehension of plant stress biology, with a particular focus on legumes and cereals. This effort ultimately contributes to bolstering sustainable agriculture and global food security. In this topic, recent advances in genomics and stress tolerance studies of cereals and legumes are presented in six publications, contributed by 52 authors.

Many complex signaling pathways are involved in the response of plants to biotic and abiotic stresses (Saijo and Loo, 2020; Zhang et al., 2022). For example, one pivotal secondary messenger in this context is calcium ion (Ca^2+^), which plays a crucial role in transmitting signals and regulating numerous physiological processes. This is accomplished through Ca^2+^ sensors and their target proteins, as highlighted by previous research (Reddy et al., 2011; Carafoli and Krebs, 2016; Edel et al., 2017). Specifically, calmodulin (CaM) and calcium-dependent protein kinase CDPKs/CPKs are ubiquitous Ca^2+^ sensors and responders in higher plants. Lv et al. identified 15 non-redundant soybean (*Glycine max*) IQM genes (a plant-specific calmodulin-binding protein) using genome-wide bioinformatics analysis and molecular biology techniques. They discussed the phylogenetic relationships, gene structure, conserved motifs, chromosome location, evolutionary pattern analysis, yeast two-hybrid analysis, and expression profile in response to abiotic stress and hormones of the soybean IQM family. Their results provide a theoretical basis for subsequent functional analysis of soybean IQM genes and insights into improving soybean resistance to biotic and abiotic stresses. Furthermore, Hu et al. indicated that the multiple members of CDPKs in wheat (*Triticum aestivum)* have diverse and vital functions in plant growth, development regulation and stress responses. They reported that every TaCDPKs member has a specific function. Their results clearly provide an important foundation for in-depth exploring the function and the signaling pathways of CDPK family members, especially the interactions between TaCDPKs and TaNOXs in wheat.

Insect pest infestation is a serious threat to cereal crops and causes extensive damage to crop production annually. The brown planthopper (BPH) is a major threat to rice production. Host-plant resistance is a key component of pest management strategies to reduce the damage caused by BPH in rice production worldwide. Xue et al. performed a research toward deep RNA sequencing to depict long non-coding RNAs (lncRNAs) profiles in rice associated with BPH feeding using the resistant Near-Isogenic Line (KW-Bph36-NIL). They elaborated insightful information on the genome-wide differentially expressed genes (DEGs) and differentially expressed lncRNAs (DELs) expression profiles of rice under BPH invasion by high throughput sequencing. They suggested that NILs could be used in BPH resistance breeding programs.

A study was undertaken by Maanju et al. to determine the genetic diversity and population structure of corn leaf aphid (CLA) *Rhopalosiphum maidis* (Fitch) resistance in barley to identify sources of resistance against *R. maidis* that considered as a common pest of cereals worldwide. In this study, 10 aphid specific simple-sequence repeats (SSR) markers were used to investigate the genetic diversity and population structure of 109 barley genotypes against *R. maidis*. Cluster analysis from the agro-morphological features grouped all the germplasm in four subpopulations and approximately 87.52% of the 109 barley genotypes shared a common major allele at any locus and finally they identified two genotypes having high resistance against CLA which highlighted their potential for inclusion in the future CLA resistance breeding programs.

With the aim of uncovering facts related to the biological mechanism underlying soybean callus induction and developmental process, Park et al. analyzed the transcriptome changes associated with the callus formation process in soybean under the influence of wounding signals and phytohormones. They identified key genes associated with transcription factors, biosynthesis, transporters, and signaling pathways related to phytohormones. The obtained results demonstrated the importance of the coordinated interplay of wounding, auxin, cytokinin, and brassinosteroid signaling pathways to activate the genes involved in determining the fate of meristematic cells. This study provides insights into the regulatory network of callus formation in soybean.

Salinity is a major environmental stress on crop productivity. Understanding the molecular and physiological mechanisms underlying salt stress tolerance will facilitate efforts to improve crop performance under salinity. In line with this aim, Wu et al. reported the analysis of the sugar beet WD40 proteins. They identified 177 bvWD40 from sugar beet and analyzed their evolutionary characteristics, protein structure, gene structure, protein interaction network and gene ontology. They concluded that *BvWD40-82* gene is a salt-tolerant candidate gene. Transgenic *Arabidopsis thaliana* seedlings expressing *BvWD40-82* showed enhanced tolerance to salt stress by elevating the contents of osmolytes and antioxidant enzyme activates, maintaining intracellular ion homeostasis and increasing the expression of genes related to salt overly sensitive (SOS) and abscisic acid (ABA) pathway. The obtained results hypothesized the importance of *BvWD40-82* as a promising candidate gene for improving crop stress resilience.

Biotic and abiotic stresses will remain major concerns for future food production and sustainability. The use of the most recent technologies in leveraging genomics, phenomics, machine learning approaches and plant biotechnology along with conventional breeding strategies is the most appropriate way to identify multifactorial interactions networks involved in plant defenses against stress conditions towards improving abiotic and biotic stress tolerance in major crops.

## Author contributions

PL: Writing – review & editing. MH: Writing – review & editing. RT: Writing – review & editing. MR: Writing – review & editing. HS: Writing – review & editing. H-JJ: Writing – review & editing.
